# A multi-level society comprised of one-male and multi-male core units in an African colobine (*Colobus angolensis ruwenzorii*)

**DOI:** 10.1371/journal.pone.0217666

**Published:** 2019-10-16

**Authors:** Samantha M. Stead, Julie A. Teichroeb

**Affiliations:** 1 Department of Anthropology, University of Toronto Scarborough, Toronto, Ontario, Canada; 2 School of the Environment, University of Toronto, Toronto, Ontario, Canada; Universidad Nacional Autonoma de Mexico Instituto de Investigaciones en Ecosistemas y Sustentabilidad, MEXICO

## Abstract

Several mammalian species exhibit complex, nested social organizations, termed multi-level or modular societies. Multi-level societies comprise stable core units that fission and fuse with one another in a hierarchical manner, forming groups that vary in size over time. Among nonhuman primates, these social systems have been confirmed in several African papionin and Asian colobine species. We use data from August 2017 to July 2018 on individually-recognized Rwenzori Angolan colobus living near Lake Nabugabo, Uganda to document the first multi-level society in an African colobine. The study band comprised up to 135 individuals organized into 12 socially and spatially distinct core units that ranged in size from 4 to 23 individuals. These core units showed a strong affinity to one another, spending roughly 75% of their time together. Core units fissioned and fused non-randomly with one another throughout the day, leading to different combinations of core units being observed. Using association indices between core units, we employed hierarchical cluster analyses and permutation tests to show that some core units associated preferentially into clans. Thus, we confirm three tiers of social organization for Rwenzori Angolan colobus: core unit, clan, and band. The social organization of this subspecies is unlike any reported previously in a nonhuman primate, with about half the core units containing a single adult male and the others containing multiple reproductive adult males. The discovery of a unique primate multi-level society in a novel lineage could allow for a better understanding of the evolution of these complex social systems across the Animal Kingdom. Preliminary data show males transfer within the band and females transfer outside of the band, which is proposed for hominin multi-level societies. This subspecies could thus also provide insight into the selective pressures underlying multi-level societies in our own lineage.

## Introduction

### Description of multi-level societies

Decades of research have been devoted to understanding the underlying components of animal social systems (mating systems, social organization, and social structure) [1–2]. Social organization, a description of group size, composition and cohesion, varies both across and within species, reflecting local ecological and social conditions [3–7]. Social systems that display short-term changes in one or more of these three parameters (size, composition, and cohesion) are said to exhibit fission-fusion dynamics [8]. In some species, fission and fusion occurs predictably along certain social boundaries. These species are described as living in multi-level (or modular) societies, which are complex social systems made up of basic social units (hereafter referred to as core units) that fission and fuse with one another in a hierarchical manner [9–10]. Core units are socially and often spatially distinct, with direct social interactions occurring most frequently between individuals in the same core unit [9]. This hierarchy of social and spatial association can extend to multiple tiers, with more complex systems showing up to four tiers of non-random association (e.g., geladas [11], hamadryas baboons [12], elephants [13]).

Multi-level societies have been described in several mammalian species, including African elephants (*Loxodonta africana*) [13–14], giraffes (*Giraffa camelopardalis*) [15], plains zebras (*Equus burchelli*) [16], prairie dogs (*Cynomys ludovicianus*) [17], khulans (*Equus hemionus*) [18], sperm whales (*Physeter microcephalus*) [19], bottlenose dolphins (*Tursiops* sp.) [20], and killer whales (*Orcinus orca*) [21]. Within the primate order, multi-level societies have been confirmed in Asian colobines (*Rhinopithecus* spp. [22–23], *Nasalis larvatus* [24]), African papionins (*Theropithecus gelada* [11], *Papio hamadryas* [25], *Papio papio* [26–28]), and humans [29–32]. Additionally, it has been suggested that uakaris (*Cacajao calvus ucayalii*) [33], drills (*Mandrillus leucophaeus*) [34], mandrills (*Mandrillus sphinx*) [35–36], and Rwenzori Angolan colobus (*Colobus angolensis ruwenzorii*) [37] live in multi-level societies; however, more conclusive data are needed.

Multi-level societies of non-human primates show exceptional group sizes, with aggregations of several hundred individuals observed at times (reviewed by Grueter and colleagues [38]). Core units are one-male/multi-female (OMU) and all-male (AMU), with no stable multi-male/multi-female core units (MMU) documented thus far [11, 22, 24–25], although see Bowler and colleagues [33] and Cui and colleagues [39] for weak evidence in uakaris and an Asian colobine (*Rhinopithecus bieti*), respectively. In African papionins, ‘follower’ or ‘secondary’ males can associate regularly with one or more OMU [26, 40–41]; however, these males rarely have sexual access to females. Up to four tiers of non-random association have been documented in African papionin (*Theropithecus gelada* [11]; *Papio hamadryas* [25], *Papio papio* [26]) multi-level societies and up to three tiers for the Asian colobines (*Nasalis larvatus* [24], *Rhinopithecus* ssp. [22, 42]).

### Evolution of multi-level societies

There are many costs and benefits of group living, mostly related to food availability [3–6, 43], predator threat [44–46] and conspecific threat [7, 47]. These factors can vary as groups move through time and space, collectively influencing optimal group size. As such, the ability to modify group size in response to fluctuating ecological and social conditions has obvious fitness benefits, which are thought to have driven the evolution of multi-level societies. Predation threat is one of several pressures thought to have selected for certain tiers of non-random association in multi-level societies, with larger groups reducing individual risk through dilution and improved predator detection [44–45, 48]. Predation by killer whales (*Orcinus orca*) has been suggested as a driver of non-random association between sperm whale core units in the Pacific Ocean [19]. Hamadryas baboon bands remained more cohesive on mornings after predator calls were heard than they typically do [49]. Low food availability, on the other hand, favours smaller groups, as this alleviates feeding competition [3–5]. Fissioning occurs at one or more tier in several multi-level societies in response to less abundant resources. During periods of lower food availability, multi-level African elephant and golden snub-nosed monkey societies fission more often, specifically at their third tier of non-random association [13, 42]. The bachelor threat hypothesis proposes that core units in multi-level societies associate so that resident males can collectively deter bachelor male(s) [16, 42, 50–52]. Associations between plains zebra one-male units (OMUs) allows stallions to more effectively deter bachelor males from approaching the females in their units [50]. Gelada males from OMUs associate more closely in the presence of bachelor males, which in geladas, form AMUs that far outnumber the resident male of a unit [53]. Males in OMUs of golden snub-nosed monkeys coordinate patrols and group defense in response to bachelor males [42, 52]. Thus, there is evidence from several species that flexible grouping in multi-level societies evolved to allow animals to optimize their group size in response to fluctuating conditions and that each tier of non-random association evolved for a specific purpose (e.g., to deter bachelor males, for predation defense, to reduce feeding competition). Since humans also form multi-level societies, the evolutionary factors that lead to their formation have been of particular interest in biological anthropology [10, 30, 38, 51].

### Rwenzori angolan colobus

To better understand the evolution of multi-level societies, it is important to first document the presence of these social systems across the Animal Kingdom. For decades, Rwenzori Angolan colobus (also referred to as Adolf Friedrich’s Angolan colobus) have been known to differ from their congeners. Early observations of this subspecies in the lowland area of Sango Bay, Uganda found large groups of about 50 individuals and multiple males within groups, which is unusual for a black-and-white colobus species [54]. This was followed by research on a population in the Nyungwe Forest in Rwanda where exceptionally large groups of about 300 were observed, with more recent data showing over 500 individuals travelling together [37, 55–56]. These group sizes are extreme outliers among black-and-white colobus (*Colobus* spp.), where groups typically average fewer than 20 individuals (reviewed by Fashing [57]). It has been suggested that this subspecies forms multi-level societies [37], but until now, concrete evidence has been lacking. Rwenzori Angolan colobus near Lake Nabugabo, Uganda form a large group of over 100 individuals that fissions into groups of varying sizes and compositions [58]. Our observations suggest that the Nabugabo Angolan colobus are organized into core units, stable sub-units of individuals that are always found together. The aims of this study were 1) to describe the sizes and compositions of core units, 2) determine if the core units associate with one another non-randomly, and if so, 3) determine the number of tiers of non-random association.

## Materials and methods

### Study site

This study was conducted at Lake Nabugabo, Masaka District, central Uganda (0°22’-12°S and 31°54’E). Nabugabo is a small lake (8.2 x 5 km) that lies just to the west of Lake Victoria at an elevation of 1,136 m. Rwenzori Angolan colobus (*Colobus angolensis ruwenzorii*) occupy patches of primary and secondary moist evergreen forest around Lake Nabugabo. We studied this subspecies in and around the Manwa Forest Reserve (~280 ha) on the west side of Lake Nabugabo, located near the trading centers of Bukumbula and Bbaale [59]. The forests that the colobus occupy are at a mean elevation of 1,151 m with a relatively flat terrain (range: 1,134–1,167 m). Annual rainfall in this area during the study period (Aug. 2017- July 2018) was 758.59 mm across two rainy seasons, one from February to May and another from September to November. The mean annual temperature was 22.2°C (min. 18.7°C, max. 26.2°C) (data from nearby Masaka, 12.5 km away). The three most dominant tree species in the forest, in terms of both stem number and basal area, are *Pseudospondias microcarpa* (Anacardiaceae), *Maesopsis eminii* (Rhamnaceae), and *Funtumia latifolia* (Apocynaceae).

### Study population and data collection

JAT began habituating a large group of over 100 Rwenzori Angolan colobus in September 2013 and follows have been conducted several days per month in most months since that time. Here, we used data collected from August 2017 to July 2018 by SMS and two field assistants where colobus were followed a mean of 11.23 days per month (range: 4–25) from approximately 7:30 am to 4:30 pm (*N* = 150 days).

Throughout the study period, one core unit was followed each observation day, with an effort made to rotate between core units throughout the month. Core units are defined as sub-units of individuals that are consistently together, resting, feeding, and moving together throughout the day. Core units are spatially distinct, meaning that an individual should typically be closer to members of its own core unit than to members of another core unit. Core unit size and composition was determined by observing individual-level proximity and behaviour. Each core unit was followed a mean of 12.5 days over the year-long study period (range: 6–17). During contact time with the colobus, we conducted focal-time follows, following one individual for two hours and taking a scan, which included: behaviour, height above the ground, characteristics of the tree occupied (species, diameter at breast height (DBH), phenology), GPS location, and the distance and ID of neighbors within 5 m. With this data collection regime, we were able to recognize all study individuals by January 2018 based on various attributes, including tail shape, broken fingers, nipple colours, and eyebrow curvature.

Scan sampling was used to record association patterns between core units [60]. Every two hours we recorded the identity of all core units that were within a 50-meter radius of the focal core unit. This two-hour time period was chosen to capture frequent changes in association among core units. A distance of 50 metres was selected as an appropriate cut-off to record two or more core units as being ‘in association’ due to three main factors: 1) Since core units were, at times, observed to be over 500 metres apart, 50 metres seemed to be a reasonable distance at which we could be sure that core units were intentionally moving into or staying in proximity of one another. 2) This distance is commonly used to define the occurrence of intergroup encounters in primates, which involve two groups coming close enough to see one another and interact [61–63]. 3) In the dense forest that our study species occupies, it was difficult to positively identify core units that were clustered beyond the units in direct association with (within 50m of) the focal unit, without leaving the focal unit.

### Data analyses

Following other researchers that have assessed the number of tiers present in multi-level societies [11, 13], we used a hierarchical cluster analysis to determine whether some core units associated preferentially with one another. We used the 2-hour scans of core unit association (*N* = 544) to calculate association indices between units. The association index that we chose was the simple ratio association index (AI) because we had good visibility during scans, and we could positively identify all core units in association with the focal unit [64]. This ratio was calculated as AI = *N*_AB_ / (*N*_A_ + *N*_B_) or the number of times that two units were in association divided by the total number of scans in which either core unit was present. For AI, a value of 0 means that core units were never observed in association on a scan and a value of 1 means that they were always together. We assessed four different clustering methods to determine which had the best fit with the data (average linkage, Ward’s weighted, complete linkage, and single linkage). The average linkage method led to the highest cophenetic correlation coefficient (CCC = 0.812), indicating that the dendrogram it produced had the best fit with the data [65], so we used this dendrogram for the rest of the analyses. To determine if there was preferential association among some core units, we counted the number of bifurcations present in the average linkage dendrogram and graphed the cumulative bifurcations to identify “knots” where the slopes below and above knots differed [66]. We assessed the significance of the slope change at knots using the Wilcoxon two-sample Z test [13]. Only one significant break was found in the cumulative distribution plot at an AI of 0.05, so we used this as the cut-off to define clans. We also computed lagged association rates, which were defined as the probability that two core units were associated given their association in the previous scan, and plotted this against null association rates. Further, we used permutation tests for preferred/avoided companionships [67] between our core units to test the null hypothesis that core unit association occurred at random. We permuted matrices of association indices and our test statistics were the coefficient of variations (CV) of the association indices (to indicate preferred associations) and the proportion of non-zero elements (to indicate avoidance between some units) [66]. We permuted matrices 1000, 2000, 5000, and 10,000 times until the p-values stabilized and we present the results from the run of 10,000 random permutations. We performed cluster analysis and permutation tests in SOCPROG v2.8 [65] and alpha values equal or less than 0.05 were deemed significant. Core units that associate preferentially were considered part of the same clan (at the calculated AI cut-off of 0.05), a term adopted from hamadryas baboon multi-level societies, where clans comprise one-male units that travel together preferentially throughout the day [25].

### Ethics approval

This research was done with the permission of the Uganda Wildlife Authority (Permit #: UWA/TDO/33/02) and the Uganda National Council of Science and Technology (Permit #: NS537). All methods were carried out in accordance with guidelines and regulations provided by the University of Toronto Animal Care Committee (Approved Protocol #: 20011416).

## Results

### Core units

We identified a total of 135 individuals organized into 12 core units that range in size from 4 to 23 individuals. Together, these 12 core units are collectively referred to as TR band, which is discussed below. The number of adult males per core unit ranged from 1 to 8 and the number of adult females ranged from 1 to 6. At the end of the study period, the 12 core units had a mean of 11.25 individuals per core unit, 2.67 adult males per core unit and 3.75 adult females per core unit. Four core units (AL, FA, MA, PO) were consistently uni-male/multi-female throughout the study period, five units were consistently multi-male/multi-female (AN, LI, LO, NE, PS), and three units changed from uni-male to multi-male or vice versa (BR, FU, PH) (Table 1).

**Table 1:** Demographic data for 12 core units of *C*. *a*. *ruwenzorii* at Nabugabo, Uganda.

Core Unit ID	Number of Individuals	Adult Females	Adult Males	Subadult Females	Subadult Males	Juveniles/Infants
**AL**	5–7	3	1	0	0	1–3
**AN**	6–9	1–3	3	1	0	1–3*
**BR**	7–8	3	1–2	1	0	2
**FA**	9	4	1	1	0	3
**FU**	8–10	2–4	1–3	1	0	2
**LI**	8–12	4	3	0	1	2–4
**LO**	18–23	6	7–8	0	3	2–6
**MA**	11–13	5	1	0	1	4–6
**NE**	13–14	5	5–6	0	0	3–4
**PH**	4–5	2	1–2	0	0	1
**PO**	9	5	1	0	0	1–4*
**PS**	14–17	4	3	1	2	4–7

All individuals were identified by January 2018 and demographic data were monitored from then until the end of data collection in August 2018.

*An infant died in this group

Core unit cohesion varied over time, with all members sitting in body contact at times and spread over a distance of up to 30m at other times. Nonetheless, members were usually closer to one another than they were to members of another core unit. Individuals within a core unit were not only spatially cohesive, but also socially cohesive; direct affiliative interactions (e.g., grooming [58], copulations, infant care) were only observed between members of the same core unit. All adult males in multi-male/multi-female core units were seen to mate with females, with the exception of one seemingly very old male in core unit PS, who sometimes trailed his unit. Core units were usually tolerant of one another and most social interactions that did occur between units were indirect (i.e., vocalizations, displaying) and typically benign, as in other primate multi-level societies [38].

### Dispersal patterns

Core units had stable memberships, with transfers between units occurring rarely. Over the study period, eight individuals were observed to transfer to and/or from core units within the TR band: four females and four males (Table 2). Two adult females transferred in parallel from another band and immigrated into TR band, joining core unit FU. In addition, another adult female immigrated into TR band from another band and joined core unit AN. One female transferred within TR band from core unit PS to core unit AN. Two males transferred in parallel within TR band from core unit FU to core unit PH. Finally, two additional transfers of adult males occurred within TR band, with one male transferring from core unit PH to core unit LO and one male transferring from core unit NE to core unit BR. In total, three individuals transferred to TR band from another band, all of which were female. All male transfers occurred within TR band. One additional female disappeared from her core unit and although she was seemingly healthy, we suspect that she may have died because she still had a juvenile offspring that nursed occasionally. In all cases where individuals were observed to leave a core unit, transfers appeared to be voluntary.

**Table 2:** Dispersal data for *C*. *a*. *ruwenzorii* at Nabugabo, Uganda.

Individual	Core Unit Left	Core Unit Joined	Month
Adult Female	Another band	AN	September 2017
Adult Female	PS	AN	November 2017
Adult Female	Another band	FU	June 2018
Adult Female	Another band	FU	June 2018
Adult Male	FU	PH	July 2018
Adult Male	FU	PH	July 2018
Adult Male	NE	BR	June 2018
Adult Male	PH	LO	May 2018

Table 2 shows the dispersal events that were documented from August 2017 until July 2018.

### Non-random associations between core units

**Fig 1:**
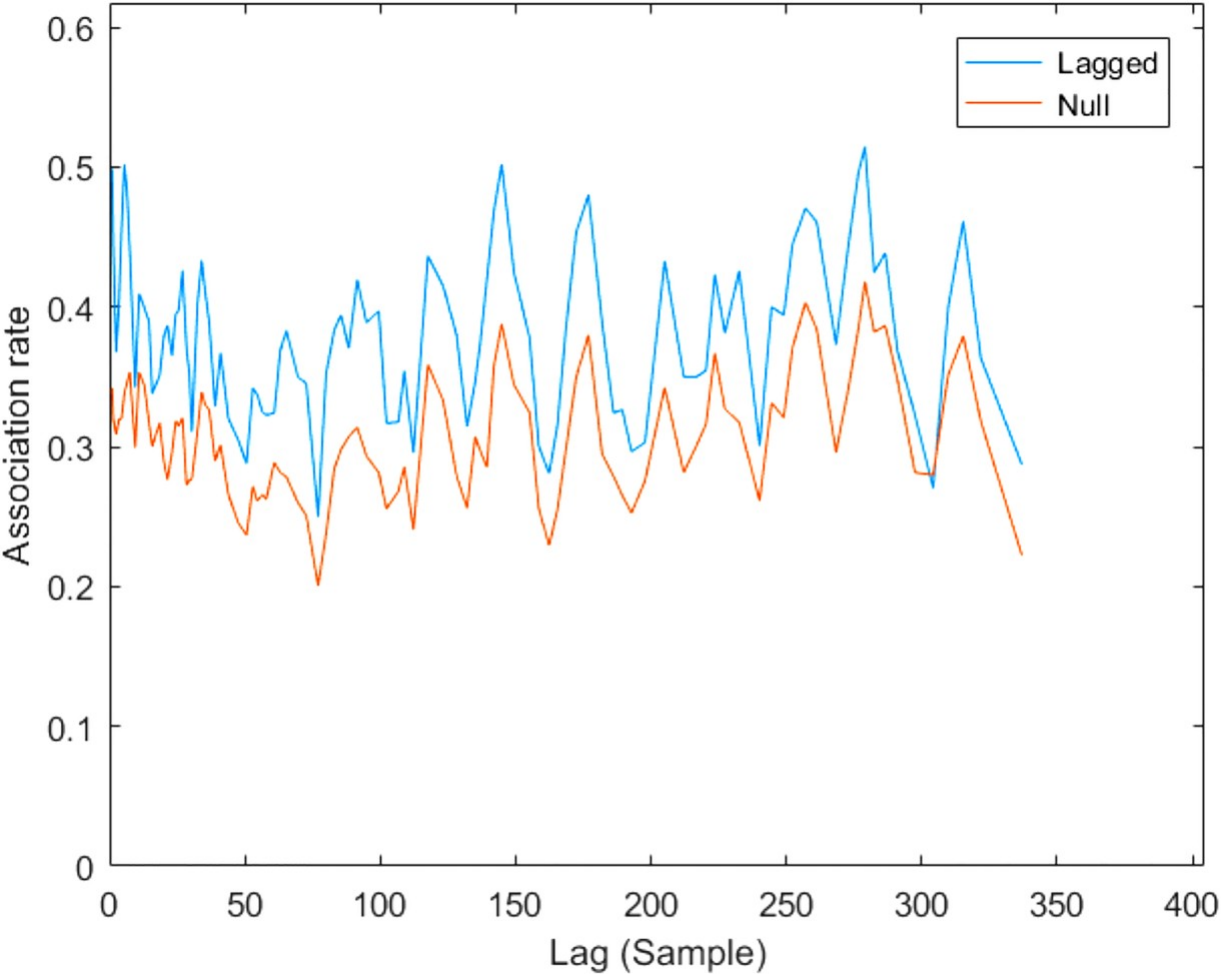
Lagged core unit association rates from 2-hour sample to 2-hour sample plotted relative to null association rates, demonstrating that there was a tendency for core units to stay in association and move together over long periods of time.

We found that focal core units were usually in association with (i.e., within 50m of) at least one of the other 12 identified core unit(s). The mean number of scans where no other core unit was in association with the focal core unit was 10.9 (±7.4 SD), for an average estimated 22.56% of time spent out of association. The mean number of core units in association with the focal core unit was 2.75 (±0.51 SD, range: 0–9). Throughout data collection, we did occasionally observe individuals that were not part of the 12 identified core units, but only rarely. Focal core units were found in association with unidentified core units (i.e., not one of the 12 described above) only three times throughout the year of data collection. We regularly observed associations between core units within TR band to last over several 2-hour scans, sometimes into the next day, and core units often moved together. This is demonstrated with lagged association rates, which show that given their association in the previous 2-hour scan, core units were consistently associated at greater than null levels (Fig 1).

**Fig 2:**
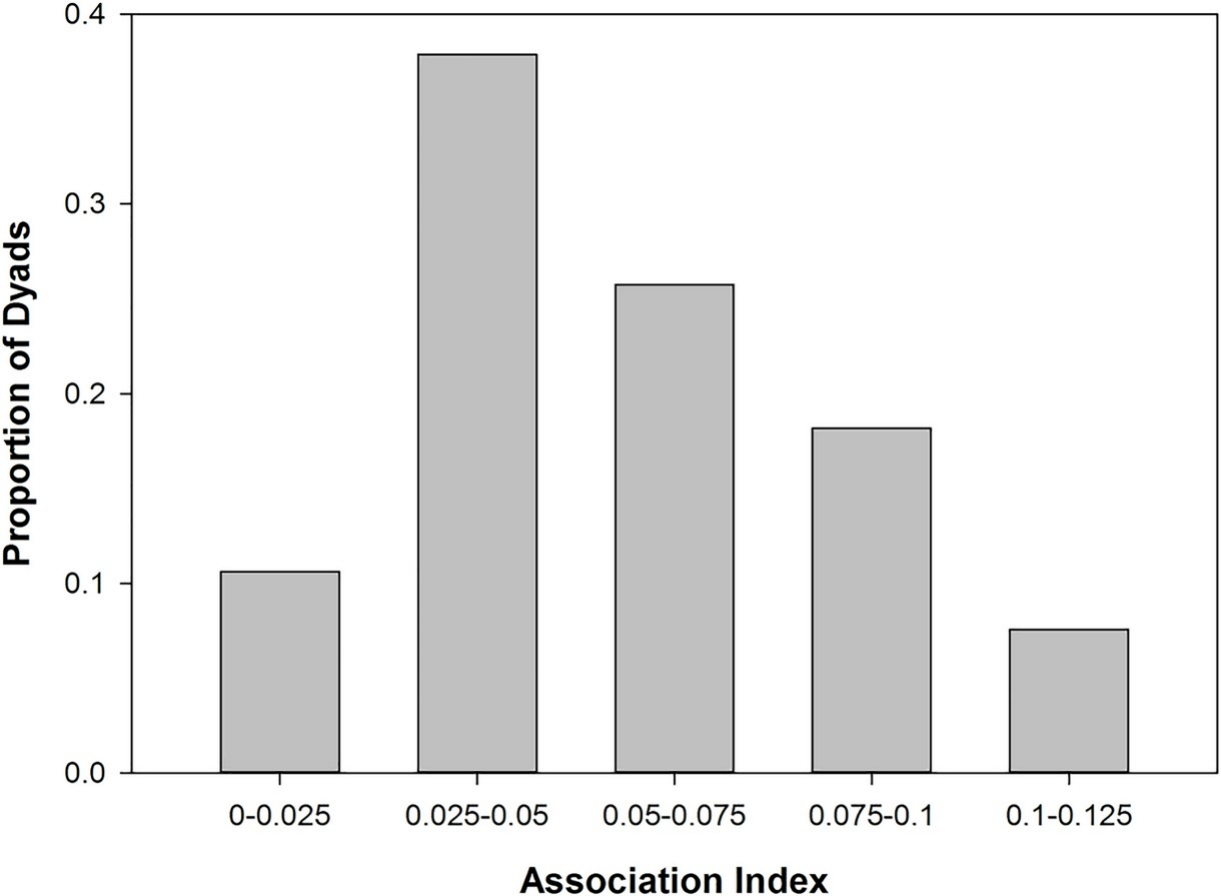
Histogram of association indices for the proportion of 66 unit dyads (12 core units) within TR band in each labeled bin. Association indices ranged from 0.0108 to 0.1206.

**Fig 3:**
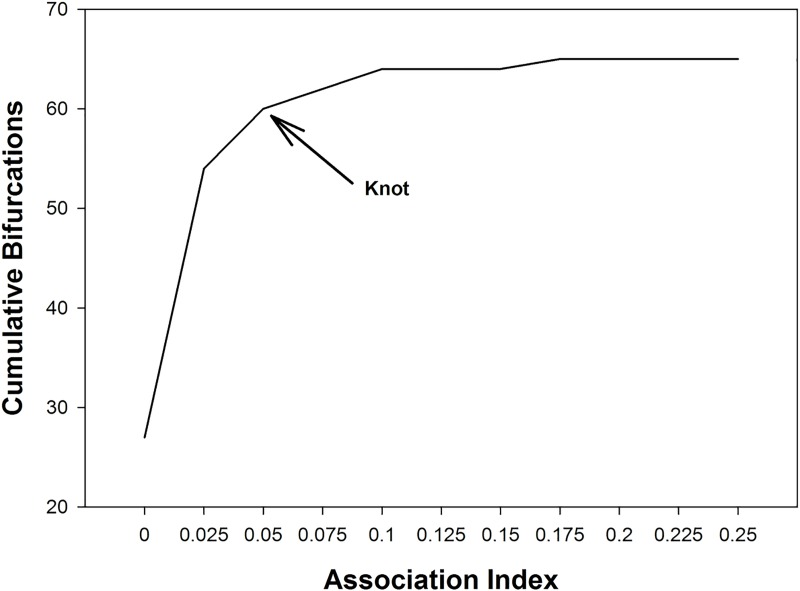
Graph of cumulative bifurcations at each increasing level of association indices for the dendrogram created using the average linkage method on core unit associations. The arrow indicates where a significant change in slope occurs (AI = 0.05), indicating a new tier of association.

**Fig 4:**
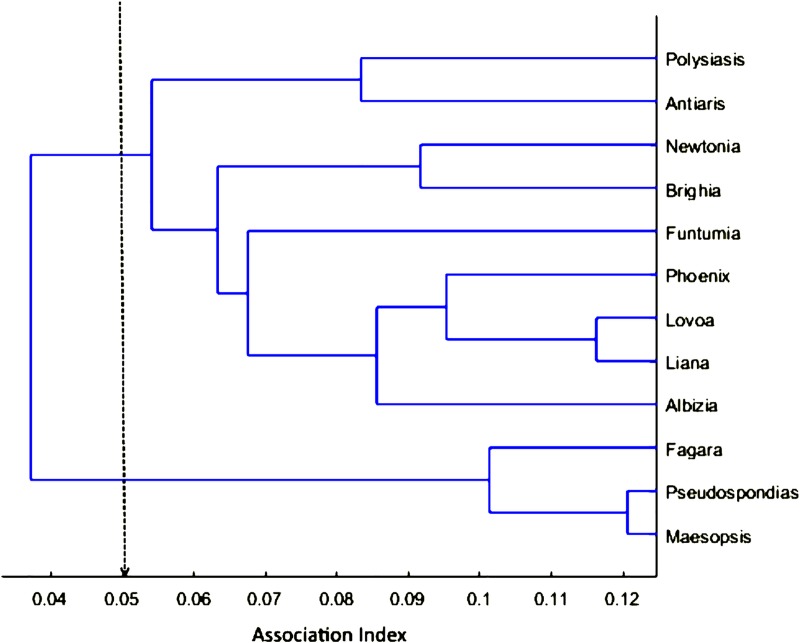
Dendrogram of core unit clusters obtained with hierarchical cluster analyses based on average linkage. Dashed line shows that at least two clans can be differentiated at an AI of 0.05.

**Fig 5:**
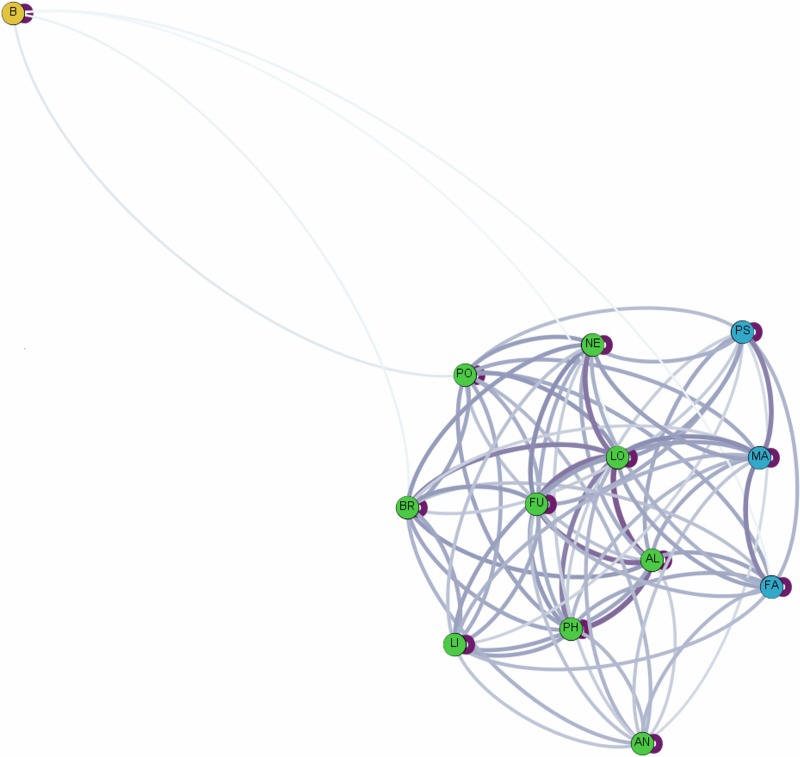
Core unit social network based on association indices between the 12 core units in TR band and neighbouring band(s) indicated by the “B”. The darkness of the lines indicates the strength of the association. Color similarity indicates units that were determined to be in the same clan using hierarchical cluster analyses.

Hierarchical cluster analyses showed that association patterns among core units of TR band were not random but rather that some core units within the band associated preferentially, making up what we refer to as a clan. Association indices, where a value of 0 means that core units were never observed in association on a scan and a value of 1 means that they were always together, ranged from 0.0108 for the core unit dyad AN-BR to 0.1206 for the core unit dyad MA-PS (Fig 2). The graph of cumulative bifurcations (Fig 3) from this dendrogram showed one significant slope change at the knot AI = 0.05 (Z-score: *Z* = 1.831, *p* = 0.034). Using this knot as a cut-off, two different clans are evident (Figs 4 and 5), one containing three core units (FA, PS, MA) and one containing nine (PO, AN, NE, BR, FU, PH, LO, LI, AL), referred to as C1 and C2 respectively. Permutation tests confirmed that observed association indices differed from random patterns of association [68]. Some units associated preferentially, as shown by the observed CV of association being significantly higher than in a random data set created with 10,000 permutations (CV_Obs_ = 0.484, CV_Rand_ = 0.44, *p* = 0.014). Additionally, some units avoided one another, in that the observed proportion of non-zero association indices was significantly lower than in the permuted random data set (Prop_Obs_ = 0.897, Prop_Rand_ = 0.96, *p* = 0.008).

## Discussion

### Tiers of non-random association

This study provides the first detailed evidence of Rwenzori Angolan colobus living in a multi-level society with three levels of non-random association: core unit, clan and band. Core units are breeding units that comprise on average 11.25 individuals that travel together, groom each other, and care for each other’s offspring. Preliminary data show core unit composition to be relatively stable, with occasional transfers by individuals of both sexes. Core units of all primate multi-level societies studied to date contain only one reproductive male. Angolan colobus at Nabugabo are thus unique in having several reproductive males in approximately half of the core units of the study band. No all-male units (AMUs) existed during the year of data presented here, or to our knowledge, in our previous observations, which were intensive by 2015. However, during the writing of this manuscript, the largest core unit (LO), which consisted of 8 adult males and 6 adult females, split permanently into two units: a OMU and a 7-member AMU.

We refer to these 12 core units collectively as TR band because they appear to have an affinity to one another. Each of the core units spent, on average, over 75% of the scans in association with one or more core unit from this band. Due to the dense forest, we were rarely able to observe all the core units in association at once; however, on several occasions, we did see all 12 core units travelling together. These core units were not spatially segregated from other Rwenzori Angolan colobus individuals. We occasionally observed unidentified individuals while walking through the forest to our target core units. However, there were only three instances during the 2-hour scans where the focal core unit was in association with unidentified individuals. Thus, other core units do live nearby; however, the study core units do not appear to have an affinity to them. Preliminary data show that the core units from TR band share a home range of about 1.5km^2^, but more data are needed to confirm this and determine how much of their home range they share with individuals from other band(s).

Core units from TR band came together (within 50m) and separated (further than 50m apart) in a variety of combinations, exhibiting fission-fusion dynamics [8]. Specifically, this population lies in Region B of Aureli’s [8] fission-fusion dynamics framework, with high variability in spatial cohesion and group size but less variability in group compositions. Core units in association had a tendency to stay together over several two-hour scans as demonstrated by lagged association rates; however, since we followed one core unit per day, our data collection regime did not allow us to determine if certain core units stayed together over even longer time periods. Our analyses found that some of the 12 core units associate more frequently than others, making up what we have termed a clan. Two different clans were delineated, one containing three core units (C1) and one containing nine (C2). This is not to say that core units from different clans are never found in association (within 50m), but rather that core units from the same clan associate more often. Essentially, TR band comprises two clans, C1 and C2, which are comprised of three and nine core units respectively. Thus, we confirm three tiers of non-random association for the Angolan colobus at Nabugabo, providing the first report of a multi-level society in an African colobine.

### Social structure and dispersal regimes

Previous work on our study population found that cross-sex bonding is stronger than bonding within each sex [58] and preliminary data show bi-sexual dispersal, a dispersal regime associated with cross-sex bonding in other primates [69]. Most research focused on social structure and dispersal regimes in multi-level societies comes from the non-human primate literature. Interestingly, social structure and dispersal regimes vary substantially across primate multi-level societies, despite a common social organization. In gelada multi-level societies, males leave their natal core unit to take over an established OMU or to join an AMU, either within the same band or another band [10–11, 70]. Female geladas remain in their natal unit, which allows for strong bonds to form amongst female kin. Conversely, hamadryas males are philopatric while females are the dispersing sex. Females are usually coerced by males to transfer from one OMU to another [30, 71]. Thus, hamadryas females do not form and maintain bonds with one another in the same way as gelada females. Instead, female hamadryas are most strongly bonded to the male in their core units (cross-bonding) [11, 72–73]. In golden snub-nosed monkeys, female-female bonds are critical for social cohesion while males are more socially peripheral [74–76]. This aligns with what is known about their dispersal patterns, as males tend to disperse from their natal band prior to sexual maturity, limiting the opportunities for this sex to form long-term bonds with other core unit members [77–79] (although see [79–80]). Interestingly, while studies on golden snub-nosed monkeys show that females transfer between OMUs of the same band, whereas males tend to disperse between bands [74, 77, 80], preliminary evidence in our population of Rwenzori Angolan colobus suggests the opposite; only females were observed to transfer from outside the band and each male transfer that occurred (*N* = 4) involved movement to and from core units within our study band. More data are needed to understand these patterns. Our preliminary observations suggest that the transfer of males between core units leads to high rates of association between those units, potentially leading to the formation of clans.

### Tier function in rwenzori angolan colobus

More data are needed to determine tier function in this multi-level society, but we can speculate by drawing on research from other animals. Predator defense, as in the case with sperm whales [19] and hamadryas baboons [49], could prove to be a tier function for either the clan or band tier in the Angolan colobus at Nabugabo. While chimpanzees [81] and leopards [82], both predators of black-and-white colobus, have been extirpated at Nabugabo, dogs kept by people pose a threat, as do snakes and birds of prey [83]. For hamadryas baboons [25], African elephants [13], and golden snub-nosed monkeys [42], research suggests that certain tiers evolved to allow groups to fine-tune their sizes in response to food availability. A similar explanation may exist for Angolan colobus clans at Nabugabo. Fusion and fission of core units is frequent in the lowland forest at Nabugabo but appears to be absent for the large aggregations of Rwenzori Angolan colobus in the montane park at Nyungwe, Rwanda [55–56, 37]. At Nyungwe, mature leaves and lichens are the predominant food sources [55, 84], but at Nabugabo, their diet consists mostly of young leaves (65%) and fruit (31%). Although both food sources are widely available, young leaves and fruit are more seasonally variable than mature leaves. This suggests that fluctuations in food availability may be important in determining the grouping patterns at Nabugabo. Our observations thus far do not suggest that bachelor males pose as much of a threat to Rwenzori Angolan colobus as they do in plains zebra [16, 50] and golden snub-nosed monkey [42, 52] multi-level societies, meaning that this may not be a tier function for this population. Future research will focus on how fluctuating selective pressures (food availability, predation threat etc.) drive changes in the grouping behaviour of Rwenzori Angolan colobus at Nabugabo.

### Evolutionary pathway

The report of a multi-level society in a new primate lineage could provide novel insight into the evolutionary pathways of multi-level societies across the Animal Kingdom, an area of study that is currently lacking. The origin of multi-level societies remains elusive and is complex due to the difficulties in teasing apart the effects of current vs. historical socioecological factors. While phylogenetic analysis is lacking for most taxa [85], it has led to two evolutionary pathways being proposed for primate multi-level societies [30, 38, 51]. Phylogenetic reconstructions of African papionins suggest that ancestral species had large multi-male/multi-female groups that underwent fissioning into present-day OMUs [30]. Fissioning is suggested to have been a response to ecological changes in the distribution of feeding sites (small and dispersed feeding patches) paired with male monopolization of females [30]. A different evolutionary route has been suggested for multi-level societies in Asian colobine species [51]. Social pressures (i.e., bachelor males) are thought to have selected for the aggregation of spatially-distinct OMUs, while widely available food is thought to have allowed for the formation of very large groups [22, 51].

Extant, closely-related species of African colobines live in either one-male/multi-female or multi-male/multi-female groups. Thus, it is possible that the evolutionary ancestors of Rwenzori Angolan colobus did as well, allowing for the aggregation of both types of core unit into a multi-level society. Large aggregations of Angolan colobus may be made possible by their diets, which rely heavily on highly abundant food, as discussed above. However, though the diet allows large groups, our data suggest that it may actually be male dispersal patterns within the band that leads to the aggregation of core units. Unlike other species of black-and-white colobus, where males are intolerant of nearby groups [86–88], the high tolerance of Angolan colobus core units in close proximity appears possible due to male-male relationships, a hypothesis that we are going to test in the coming years.

### Potential importance to human evolution

Nonhuman primate multi-level societies are often compared to human social organizations [89–91]. Human societies are typically multi-level (or multi-family) groups comprised of families that associate non-randomly to form bands, with the majority of interacting families being monogamous pairs (with some polygynous families). Though humans show flexible dispersal patterns [92], female marriage outside the band is a common human pattern that usually occurs with strong male relationships [93–94]. Unlike other known primates, people maintain close bonds with their natal kin even after they leave to breed elsewhere [95]. These lifelong bonds among dispersed individuals allow extended networks of kin in humans [89, 96]. They also lead to multi-family groups that are strongly bonded and with high levels of coordination between-groups [90].

We are only beginning to understand the unique multi-level society shown by Rwenzori Angolan colobus, but it appears that it shows several important features that evolved in our own lineage. Female dispersal beyond the band is rare in nonhuman primate multi-level societies, only described previously for hamadryas baboons [30], and this may make Rwenzori Angolan colobus important in offering insight into human evolution. In addition, male tolerance between- and within-core units appears to be important to the formation of this multi-level society and may be due to male kinship within the band, a hypothesis we plan to test in the future. If male alliances persist after dispersal, leading to core units grouping into clans, this could tell us more about how extended networks of bonds evolved in our own lineage.

## Supporting information

S1 Dataset Core unit associations. (XLSX)

S2 Dataset GPS points. (XLSX)
